# Cryptotanshinone Is a Intervention for ER-Positive Breast Cancer: An Integrated Approach to the Study of Natural Product Intervention Mechanisms

**DOI:** 10.3389/fphar.2020.592109

**Published:** 2021-01-11

**Authors:** Huayao Li, Chundi Gao, Qing Liang, Cun Liu, Lijuan Liu, Jing Zhuang, Jing Yang, Chao Zhou, Fubin Feng, Changgang Sun

**Affiliations:** ^1^ College of Chinese Medicine, Shandong University of Traditional Chinese Medicine, Jinan, China; ^2^ College of First Clinical Medicine, Shandong University of Traditional Chinese Medicine, Jinan, China; ^3^ Department of Basic Medical Sciences, School of Medicine, Xiamen University, Xiamen, China; ^4^ Departmen of Oncology, Weifang Traditional Chinese Hospital, Weifang, China; ^5^ Department of Oncology, Affilited Hospital of Weifang Medical University, Weifang, China; ^6^ Department of Basic Medical Science, Qingdao University, Qingdao, China; ^7^ Chinese Medicine Innovation Institute, Shandong University of Traditional Chinese Medicine, Jinan, China

**Keywords:** CPT, breast cancer, estrogen receptor, high-throughput data, proliferation inhibition

## Abstract

**Background: **Resistance to endocrine therapy has hampered clinical treatment in patients with ER-positive breast cancer (BRCA). Studies have confirmed that cryptotanshinone (CPT) has cytotoxic effects on BRCA cells and can significantly inhibit the proliferation and metastasis of ER-positive cancer cells.

**Methods: **We analyzed the gene high-throughput data of ER-positive and negative BRCA to screen out key gene targets for ER-positive BRCA. Finally, the effects of CPT on BRCA cells (MCF-7 and MDA-MB-231) were examined, and quantitative RT-PCR was used to evaluate the expression of the key targets during CPT intervention.

**Results: **A total of 169 differentially expressed genes were identified, and revealed that CPT affects the ER-positive BRCA cells by regulating *CDK1*, *CCNA2*, and *ESR1*. The overall experimental results initially show that MCF-7 cells were more sensitive to CPT than MDA-MB-231 cells, and the expression of *ESR1* was not affected in the BRCA cells during CPT intervention, while the expression of *CDK1* and *CCNA2* were significantly down-regulated.

**Conclusion: **CPT can inhibit the proliferation and migration of BRCA cells by regulating *CDK1*, *CCNA2*, and *ESR1*, especially in ER-positive BRCA samples. On the one hand, our research has discovered the possible mechanism that CPT can better interfere with ER+ BRCA; on the other hand, the combination of high-throughput data analysis and network pharmacology provides valuable information for identifying the mechanism of drug intervention in the disease.

## Introduction

Estrogen receptor (ER) status has been shown to have prognostic and therapeutic implications in breast cancer (BRCA) patients. Estrogen binds to its nuclear receptor and triggers gene regulation, transcription of downstream genes, and participates in mechanisms of disease development. In addition, the binding of estrogen to its membrane receptor can mediate rapid non-genomic effects, affecting protein expression by regulating the transcriptional function of second messengers (Yip and Rhodes, 2014), and it is therefore evaluated and guides the standard treatment for all newly diagnosed invasive, recurrent, and metastatic BRCA cases (Jorns, 2019). Clinically, ER status is usually assessed by immunohistochemical staining to assess protein expression. Currently, selective estrogen receptor modulators and aromatase inhibitors are used alone, or in combination with chemotherapy to treat ER-positive BRCA, which can significantly improve the survival rate of estrogen receptor-positive BRCA patients (Mazumdar et al., 2019). However, the resistance to endocrine therapy drugs hinders the clinical treatment of ER-positive BRCA, and new endocrine therapy drugs need to be explored to overcome this challenge.

Cryptotanshinone (CPT) is a diterpenoid derived from the dried roots and rhizomes of Salvia miltiorrhiza. It has been used to treat chronic obstructive pulmonary disease, BRCA, and inflammatory diseases (Ruan et al., 2019). CPT has good solubility and permeability, and has a variety of pharmacological activities, including anti-inflammatory, anti-proliferative, and anti-infective (Bai et al., 2019; Zhang et al., 2019). CPT inhibits epithelial-mesenchymal transition *via* the transforming growth factor β/Smads signaling pathway, regulates pathological epithelial-mesenchymal transition, and promotes angiogenesis and recovery of normal organ function (Avila-Carrasco et al., 2019). In the treatment of tumors, CPT has a cytotoxic effect, and dual ability to simultaneously inhibit tumor cell proliferation and promote anti-tumor immunity (Han et al., 2019). Studies have shown that CPT has a cytotoxic effect in BRCA cells and can promote tumor cell apoptosis through the JAK/STAT and Ros signaling pathways (Zhou et al., 2014; Vundavilli et al., 2019). CPT significantly inhibits the proliferation of ERα-positive cancer cells, while ERα-negative cancer cells are not sensitive to CPT (Pan et al., 2017). A study of the mechanism underlying this difference by Li et al. revealed that CPT can effectively inhibit estrogen-induced ER transactivation and expression of ER target genes, and concluded that CPT inhibits the growth of BRCA cells in an ERα-dependent manner (Li et al., 2015). Pan et al. proposed that CPT inhibits mTOR signaling in MCF-7 cells but does not inhibit mTOR signaling in MDA-MB-231 cells. The inhibition of mTOR by CPT is dependent on ERα in BRCA (Pan et al., 2017). Park et al. found that CPT could sensitize a variety of anticancer agents (including Fas/Apo-1, TNF-α, cisplatin, etoposide and 5-FU) by inducing ER stress (Park et al., 2012). Therefore, in this study, we evaluated the pharmacological effects of CPT on MCF-7 and MDA-MB-231 cells, and investigated its intrinsic mechanism of action to justify its use in the clinical treatment of ER-positive BRCA.

In the post-genome era, with the widespread use of next-generation sequencing technology, clinical and genomic information of patients are comprehensively analyzed and stored (Barrett et al., 2013; Tomczak et al., 2015). A new field of research, namely, “Bioinformatics Gene Big Data System Analysis” has evolved, that analyzes the large amounts of stored data to further explore the molecular mechanism of disease development. It involves the integration of molecular signaling and regulation of key cell functions to obtain a theory, i.e., instructive for the clinical treatment of diseases.

In this study, we determined the inhibitory effect of CPT on the BRCA cells, MCF-7 and MDA-MB-231, and the overall experimental data initially showed that MCF-7 cells are more sensitive to CPT. We found that CPT inhibits the proliferation of BRCA cells by interfering with the expression and signal regulation of *cyclin-dependent kinase 1 (CDK1), estrogen receptor (ESR1),* and *cell cycle egg A2 (CCNA2)*. These data support CPT as a potential drug for the treatment of BRCA.

## Materials and Methods

### Data Source and Processing

The Gene Expression Omnibus (GEO) database is a high-throughput chip expression profiling database that can be retrieved to obtain high-throughput data, that effectively complements traditional laboratory data resources. In this study, two chips consisting of ER-positive and ER-negative BRCA-related data were selected from the GEO database. Among them, the chip GSE31192, based on the GPL570 (HG-U133_Plus_2) Affymetrix Human Genome U133 Plus 2.0 Array platform annotation (Harvell et al., 2013), consisted of 18 ER-positive samples and 15 ER-negative samples, and the chip GSE43837 annotated by the GPL1352 (U133_X3P) Affymetrix Human X3P Array platform (McMullin et al., 2014), consisted of eight ER-positive samples and 30 ER-negative samples. DEGs were obtained by analyzing the gene expression profiles using the GEO2R tool for further investigation.

### Analysis of Differentially Expressed Genes and Construction of PPI Networks

The David database (https://david.ncifcrf.gov/) (Jiao et al., 2012) provides a comprehensive set of functional annotation tools to understand the biological significance of a large number of genes, which can be used for gene ontology (GO) and Kyoto Gene and Genomic Encyclopedia (KEGG) analysis of DEGs. *p* < 0.05 was used as a cut-off condition for screening GO and KEGG pathway enrichment analysis. In addition, to analyze the interaction between the DEGs, the protein interaction network of DEGs was constructed using String database (https://string-db.org/cgi/input.pl) (Szklarczyk et al., 2015) and Cytoscape 3.5.1 software (Shannon et al., 2003). The topology and module analysis of the PPI network was performed through CytoNCA and MCODE plug-in, respectively. Finally, the key nodes in the PPI network were identified, including the main DEGs that could be used as a biomarker of ER-positive BRCA.

### Molecular Docking

Molecular docking is an important method to realize structure-based drug design by studying the interaction between small ligand molecules and receptor biomolecules and predicting their affinity. In this study, to screen the key DEGs for further studies, the Surflex-Dock program interfaced with Sybyl X.0 was utilized to dock CPT with DEGs. Surflex-Dock is a fully automated and flexible docking procedure for ligands. It relies on rigid receptor approximation to simulate ligand-receptor binding patterns, and evaluate docking simulation results by docking score (Jain, 2007). Firstly, the structure of CPT was constructed by ChemDraw Ultra 12.0 software and optimized in Sybyl X (Cousins, 2011). Then, the X-ray crystal structure of DEGS was extracted from RCSB protein database (http://www.rcsb.org/). The eutectic ligands and structural water molecules were removed from the crystal structure before the start of the docking simulation. Add polar hydrogen atoms and distribute Coleman’s total atomic charge to protein atoms. In our study, the ligand model is based on considering the structural similarity of the co-crystallized ligand and the target compound, and the ProtoMol extension and ProtoMol threshold parameters are set to the default values of 0 and 0.50, respectively. Subsequently, the process of molecular docking was simulated. Finally, the DEGs that could be docked with CPT were identified as the key DEGs for further experimental verification.

### Effect of CPT on Cell Viability

To determine the effect of CPT on the activity of BRCA cells, ER-positive (MCF-7) and ER-negative BRCA cells (MDA-MB-231) were inoculated in 96-well culture plates at the density of 1 × 10^3^ cells/well and cultured for 24 h at 37°C, 5% CO_2_ level, constant pH (pH: 7.2–7.4), and high relative humidity (95%). The cells were then exposed to 0, 1, 5, or 10 mg/ml of CPT solution (98% purity, purchased from Shanghai Yingxin Co., Ltd. China) and cultured for 0, 24, 48, 72, and 96 h, respectively. Fresh medium containing 10% CCK-8 (WST-8, Yiyuan Biotechnology) (Gao et al., 2018) was used to replace the culture medium, and the cells were further cultured for 4 h under the same conditions. Finally, the absorbance was read at 490 nm using a scanning porous spectrophotometer (Thermo Scientific, China). The effect of CPT on cell activity was described by cell proliferation rate (%). The formula used was as follows:

Cell proliferation rate(%)=(ODCPT−ODBlank)/(ODCPT−ODBlank)×100%

### Cell Migration

The cells were inoculated in six-well culture plates at the concentration of 1 × 10^5^ cells/ml. A marker pen was used to draw three straight lines on the back of the six-well plate, and then the cells were cultured overnight as monolayer cells under the culture conditions described earlier. The next day, two straight marks (scratches) were drawn across the monolayer with a 200 μL pipette tip as evenly as possible perpendicular to the horizontal line on the back. The wells were washed twice with PBS to remove the suspended cells and fresh serum-free medium was added. The cells in the wells were then exposed to the CPT at 0, 1, 5, and 10 mg/ml, and cultured for 24 h. Finally, the distance of cell movement affected by different concentrations of CPT solution was measured and photographed using an inverted microscope (100×), and the migration was calculated.

### Transwell Invasion Test

The cell concentration was adjusted to 1 × 10^5^ cells/ml in DMEM medium. Then 100 μL MCF-7 and MDA-MB-231 cells were inoculated into transwell migration chambers. Medium containing CPT at a concentration of 0, 1, 5, or 10 mg/ml was added to the lower chamber. The cells were cultured for 24 h under the culture conditions described earlier. The migration chamber was removed, the cells in the upper layer of the filter membrane were removed with cotton swabs and the membrane was fixed with methanol for 20 min. After drying at room temperature, the cells were stained with crystal violet for 20 min. The number of cells that had passed through the membrane in five different fields of vision were counted under 100× using a light microscope, and the inhibitory effect of CPT on the invasion of MCF-7 and MDA-MB-231 cells was calculated.

### Quantitative RT-PCR Determination of Key DEGs

MCF-7 and MDA-MB-231 cells at a concentration of 1 × 10^5^ cells/ml in a volume of 6.0 ml were seeded in a cell culture dish (*d* = 100 mm), and cultured under the set conditions for 24 h. The cells were then exposed to 0, 5, and 10 mg/ml CPT and cultured for 24 and 48 h. Total RNA was extracted from the MCF-7 and MDA-MB-231 cells using TRIzol (Vazyme, Nanjing, Jiangsu), according to the manufacturer’s instructions, and stored for use after precipitation, cleaning, drying and resuspension. Finally, cDNA was synthesized by reverse transcription using Transciptor First Strand cDNA synthesis package (Vazyme). The primer sequences used were as follows: 5′-AGC​CGC​CCT​TTC​CTC​TTT-3′ (forward) and 5′-AAC​CCC​TTC​CTC​TTC​ACT​TTC​TA-3′ (reverse) for CDK1; 5′-CTC​GGC​CCT​GCG​TGG​TCT​CG-3′ (forward) and 5′-GCG​CTG​CCT​TTT​CCG​GGT​TGA​TA-3′ (reverse) for CCNA2; 5′-CCT​CTA​ACC​TCG​GGC​TGT​GCT​CT-3′ (forward) and 5′-GCC​GCG​GCG​TTG​AAC​TCG​TAG​G-3′ (reverse) for ESR1.

### Statistical Analysis

The experimental results are presented as mean ± SD. The statistical and quantitative analysis of the data was visualized using GraphPad Prism 6.0 (GraphPad Software Inc., La Jolla, CA), Image J (National Institutes of Health, United States), and the SPSS software (IBM). The one-way analysis of variance (one-way ANOVA) and Kruskal-Wallis test were used to determine the difference between the groups, and *p* < 0.05 was used as the cut-off for statistical significance.

## Results

### Identification of Differentially Expressed Genes Between ER-Positive and ER-Negative BRCA

**FIGURE 1:**
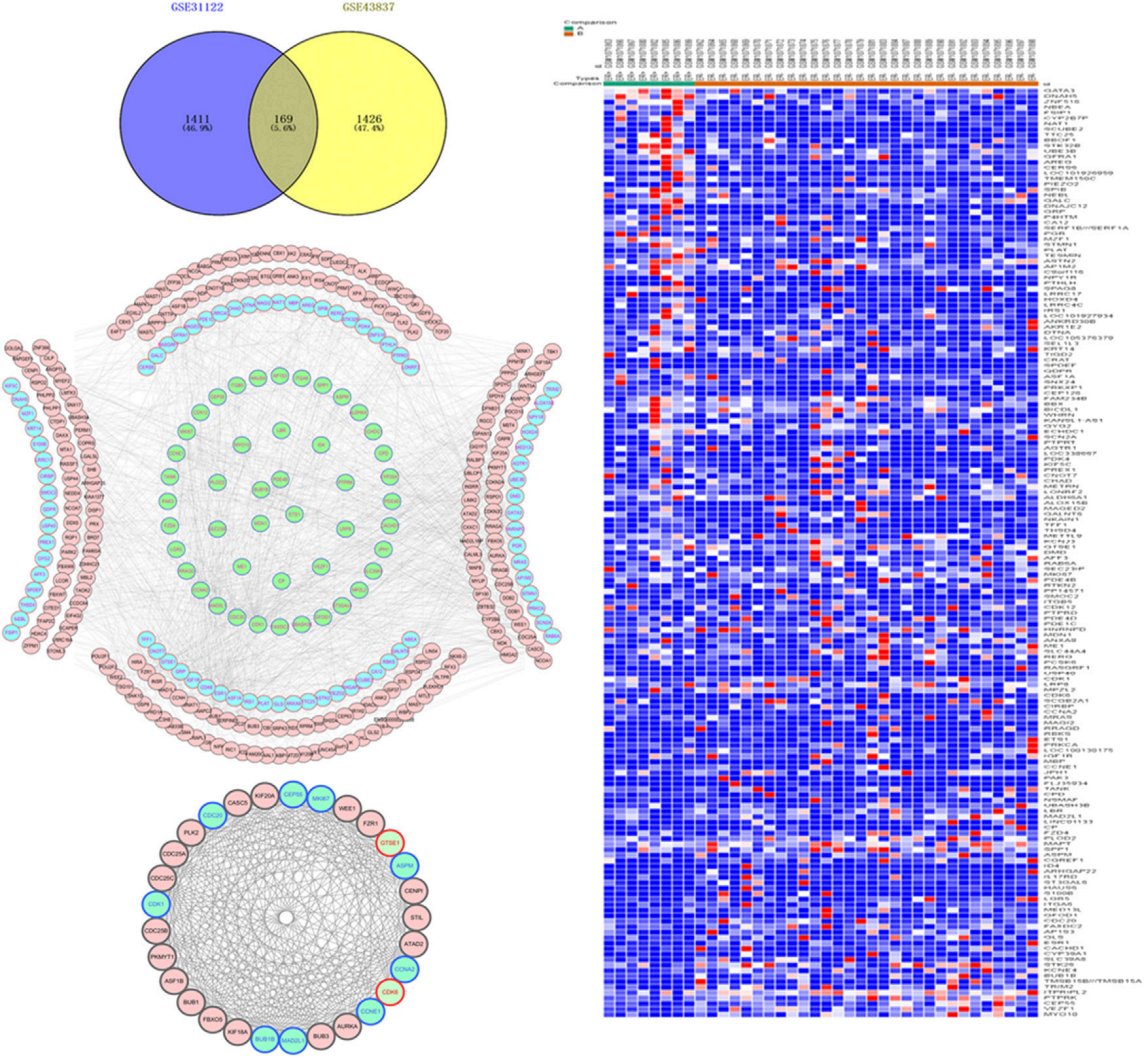
Acquisition of differentially expressed genes (DEGs) and construction of protein interaction network in estrogen receptor (ER)-positive and ER-negative breast cancer. **(A)** Overlapping 169 DEGs were identified from two microarray data profiles (GSE31192, GSE43837). Blue represents 2,153 DEGs identified from GSE31192 chip, and yellow represents 1,689 DEGs identified from GSE43837 chip. The intersection areas represent 169 common DEGs. **(B)** Based on the GSE43837 chip data composed of eight ER positive samples and 30 ER negative samples, Heat maps of 169 DEGs are drawn. The color from blue to red shows a trend from low expression to high expression. Protein interaction network construction and module analysis of 169 DEGs, 169 DEGs related genes were also included in the mapping. **(C)** The protein interaction network of 169 genes, including 334 nodes and 1,298 edges. **(D)** The most significant module in the network, including 29 nodes and 306 edges. Blue represents the down-regulated DEGs, green represents the up-regulated DEGs, and red represents the 169 DEG-related genes obtained in String.

Based on GEO2R analysis, with |LogFC| > 1 and *p* < 0.05 set as the cut-off conditions, 2,153 DEGs were identified in the chip GSE31192, and 1,689 DEGs were identified in the chip GSE43837. To ensure the reliability of the results, 169 genes identified by both chips were used as the DEGs between ER-positive and ER-negative BRCA (Figure 1A). This set included 59 up-regulated genes and 110 down-regulated genes (Figure 1B).

### Function Evaluation of DEGs and Construction of PPI Network

To further explore the functional abnormalities that may be caused by the DEGs in the human body, the DEGs were analyzed by GO and KEGG using the DAVID database. The enrichment results of KEGG pathways (Table 1) show that with *p* < 0.05 as the cutoff condition, these DEGs are mainly involved eight pathways, including cell cycle, progesterone-mediated oocyte maturation, PI3K-Akt signaling pathway, and p53 signaling pathway. The GO analysis identified DEGs that were particularly abundant in the classification of molecular functions, biological processes, and cellular components (Table 2). In the biological process (BP) group, DEGs were mainly enriched in negative regulation of cell cycle and cell proliferation, signal transduction, cell division and so on. The molecular functions (MF) group was mainly enriched in various combinations, such as cyclin binding, and cyclin-dependent protein serine/threonine kinase activity. In addition, the components in the cell component (CC) group were mainly related to cell periphery, cytosol, and *trans*-Golgi network membrane.

**TABLE 1:** KEGG pathway enrichment analysis of 169 DEGs (*p* < 0.05).

KEGG-term	Genes	*p* value
Cell cycle	*CCNE1, CDK1, MAD2L1, BUB1B, CDK6, CDC20, CCNA2*	1.14E-03
Focal adhesion	*PRKCA, IGF1R, ITGA6, RASGRF1, PAK3, ITGB5, SPP1, CHAD*	3.40E-03
Oocyte meiosis	*PGR, CCNE1, CDK1, IGF1R, MAD2L1, CDC20*	4.09E-03
Progesterone-mediated oocyte maturation	*PGR, CDK1, IGF1R, MAD2L1, CCNA2*	9.48E-03
Morphine addiction	*PRKCA, PDE1C, PDE4B, PDE4D, KCNJ3*	1.11E-02
PI3K-Akt signaling pathway	*PRKCA, CCNE1, IGF1R, ITGA6, ITGB5, CDK6, IRS1, SPP1, CHAD*	1.67E-02
p53 signaling pathway	*CCNE1, CDK1, CDK6, GTSE1*	2.61E-02
Proteoglycans in cancer	*PRKCA, IGF1R, MRAS, ESR1, ITGB5, FZD4*	4.23E-02

**TABLE 2:** Based on the GSE43837 chip data composed of eight ER positive samples and 30 ER negative samples, Heat maps of 169 DEGs are drawn.

Category	Term	Count	*p* value
BP	Signal transduction	26	1.32E-05
	Negative regulation of cell cycle	5	2.61E-04
	Negative regulation of cell proliferation	11	2.00E-03
	Neuron projection development	5	1.03E-02
	Regulation of ryanodine-sensitive calcium-release channel activity	3	1.11E-02
	Oxidation-reduction process	12	1.19E-02
	Negative regulation of relaxation of cardiac muscle	2	1.68E-02
	Mitotic nuclear division	7	1.89E-02
	Phosphatidylinositol 3-kinase signaling	3	2.17E-02
	Negative regulation of ubiquitin-protein ligase activity involved in mitotic cell cycle	4	2.23E-02
	Positive regulation of ubiquitin-protein ligase activity involved in regulation of mitotic cell cycle transition	4	2.66E-02
	Cellular response to estradiol stimulus	3	2.81E-02
	Anaphase-promoting complex-dependent catabolic process	4	2.94E-02
	Cell division	8	2.94E-02
	Brown fat cell differentiation	3	2.98E-02
	Epithelial cell proliferation involved in mammary gland duct elongation	2	3.34E-02
	Regulation of erythrocyte differentiation	2	3.34E-02
	Protein ubiquitination involved in ubiquitin-dependent protein catabolic process	5	4.10E-02
	Positive regulation of cell proliferation	9	4.45E-02
	Positive regulation of DNA replication	3	4.91E-02
MF	Cyclin binding	3	1.19E-02
	3′,5′-cyclic-nucleotide phosphodiesterase activity	3	1.56E-02
	ATPase binding	4	2.40E-02
	Cyclin-dependent protein serine/threonine kinase activity	3	3.26E-02
	Iron ion binding	5	3.95E-02
	Structural constituent of muscle	3	4.80E-02
CC	Cell periphery	4	3.80E-03
	Cytosol	41	4.38E-03
	Centrosome	10	7.76E-03
	*trans*-Golgi network membrane	4	2.96E-02
	Cell surface	10	3.18E-02
	Clathrin-coated endocytic vesicle membrane	3	4.32E-02

The color from blue to red shows a trend from low expression to high expression.

To further analyze the interaction between the DEGs, we used the String database to mine for related genes, construct the PPI network and visualize it through the Cytoscape software (Figure 1C). Then the plug-in MCODE was used to analyze the module of the PPI network, and the most meaningful module was determined based on the score. The module consisted of 29 nodes and 306 edges (Figure 1D). The KEGG pathway enrichment analysis showed that the DEGs in the module were mainly enriched in the cell cycle, p53 signaling pathway, oocyte meiosis, microRNAs in cancer, etc. Since the nodes with high connectivity play an important role in maintaining the whole PPI network structure, and are an indispensable part of the network, we analyzed the topology of DEGs through the plug-in CytoNCA, and used degree >50 as the screening condition. Three nodes with high connectivity were identified from the PPI network, which were *CDK1*, *ESR1*, and *CCNA2*, respectively. These three genes were the most important nodes in the network and were considered to be the key DGEs between ER-positive and ER-negative BRCA.

### Construction of Molecular Docking Model

**FIGURE 2:**
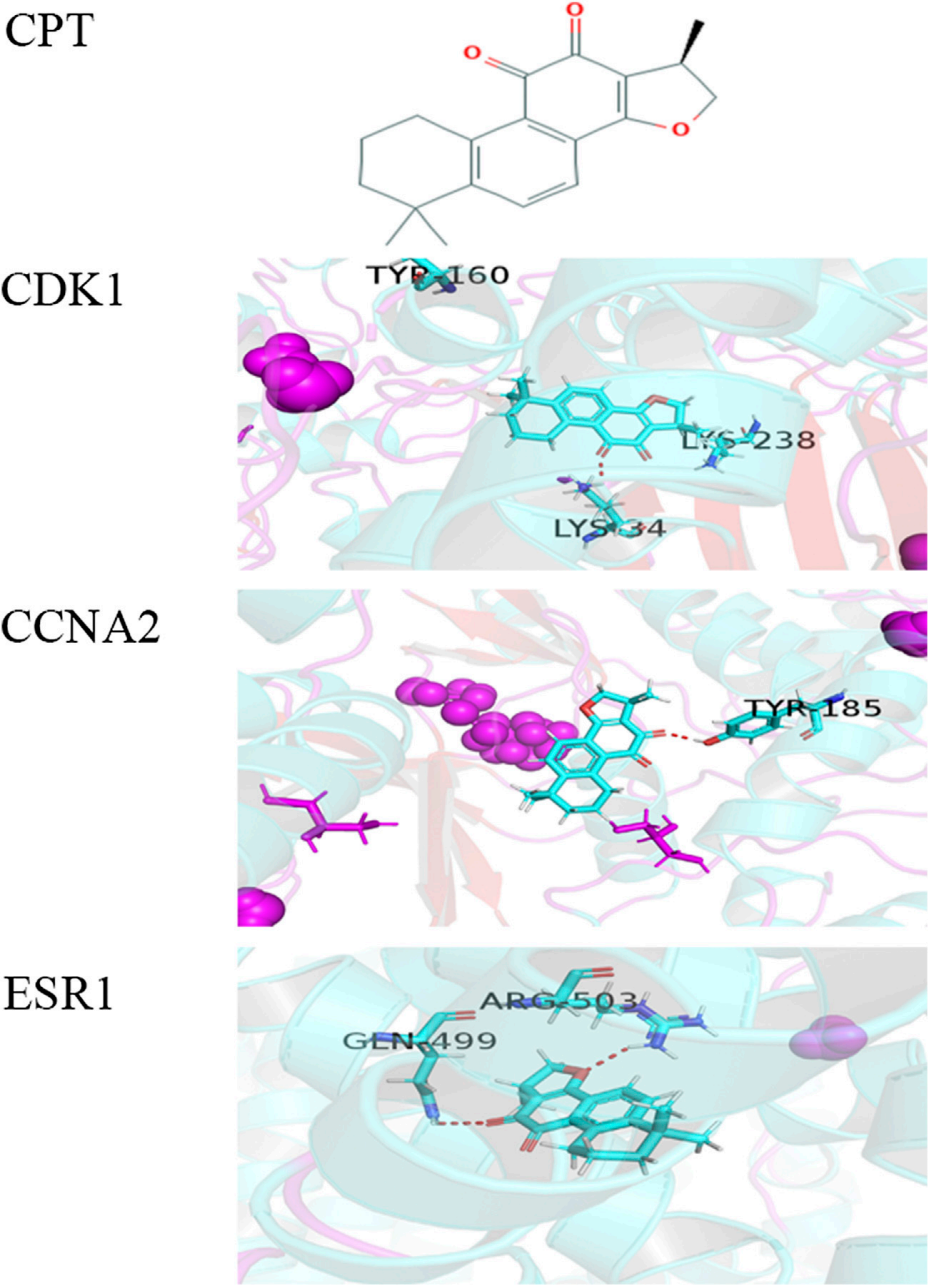
The molecular docking model between CPT and three key DEGs. **(A)** Chemical structure of CPT: (R)-1,2,6,7,8,9-Hexahydro-1,6,6-trimethyl-phenanthro(1,2-b)furan-10,11-dione.the docking scores of *CDK1* **(B)**, *CCNA2* **(C)**, and *ESR1* **(D)** were 4.3928, 4.1092, and 4.4081. The red dashed line represents the docking hydrogen bond between CPT and three key DEGs.

To further explore the mechanism of interaction between CPT and three key DEGs, we constructed the molecular docking model between CPT and key DEGs by Sybyl X software. The docking results showed that the docking scores between the three kinds of DEGs and CPT were higher than four, in which the docking scores of *CDK1*, *ESR1*, and *CCNA2* were 4.3928, 4.4081, and 4.1092, respectively. The results showed that the three key DEGs had a good binding effect with CPT, and there were stable binding sites in the small molecular model of CPT (Figure 2). This is a predictive study, Based on this, the three genes docked with CPT can be identified as key DEGs for further experimental verification.

### Effects of CPT on Viability, Migration, and Invasion of MCF-7 and MDA-MB-231 Cells in BRCA

**FIGURE 3:**
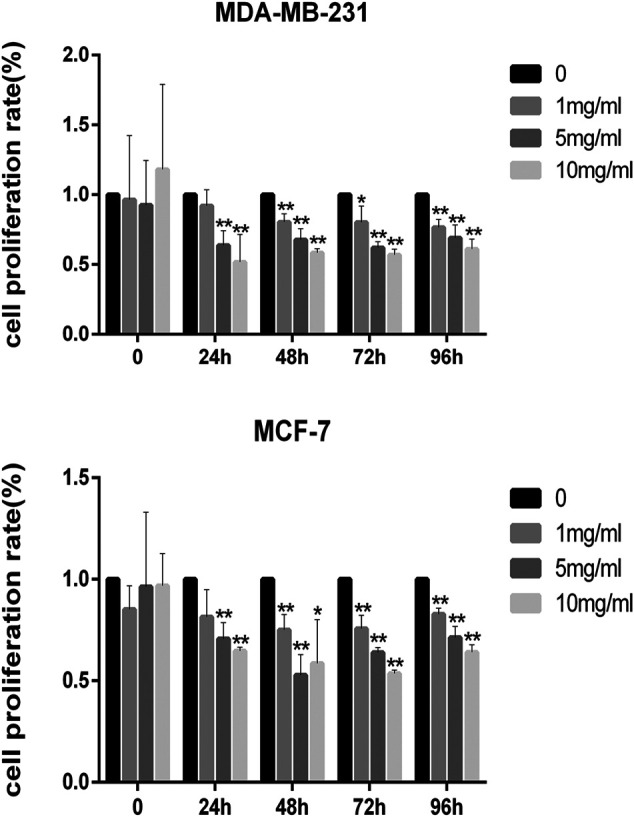
All concentrations of CPT inhibited the rate of proliferation of MDA-MB-231 **(A)** and MCF-7 **(B)** BRCA cells. *p < 0.05, **p < 0.01 vs. control (0 mg/ml).

The results of the cell viability experiment showed that all concentrations of CPT inhibited the rate of proliferation of MCF-7 and MDA-MB-231 BRCA cells (Figure 3), and the inhibitory effect of CPT was both time- and concentration-dependent. In addition, by comparing the cell proliferation rate at different time points, we found that CPT showed the highest inhibitory effect at 48 h, and at a concentration of 5 mg/ml in MCF-7 cells. However, the effect of CPT on the proliferation rate of MDA-MB-231 cells was not regular.

**FIGURE 4:**
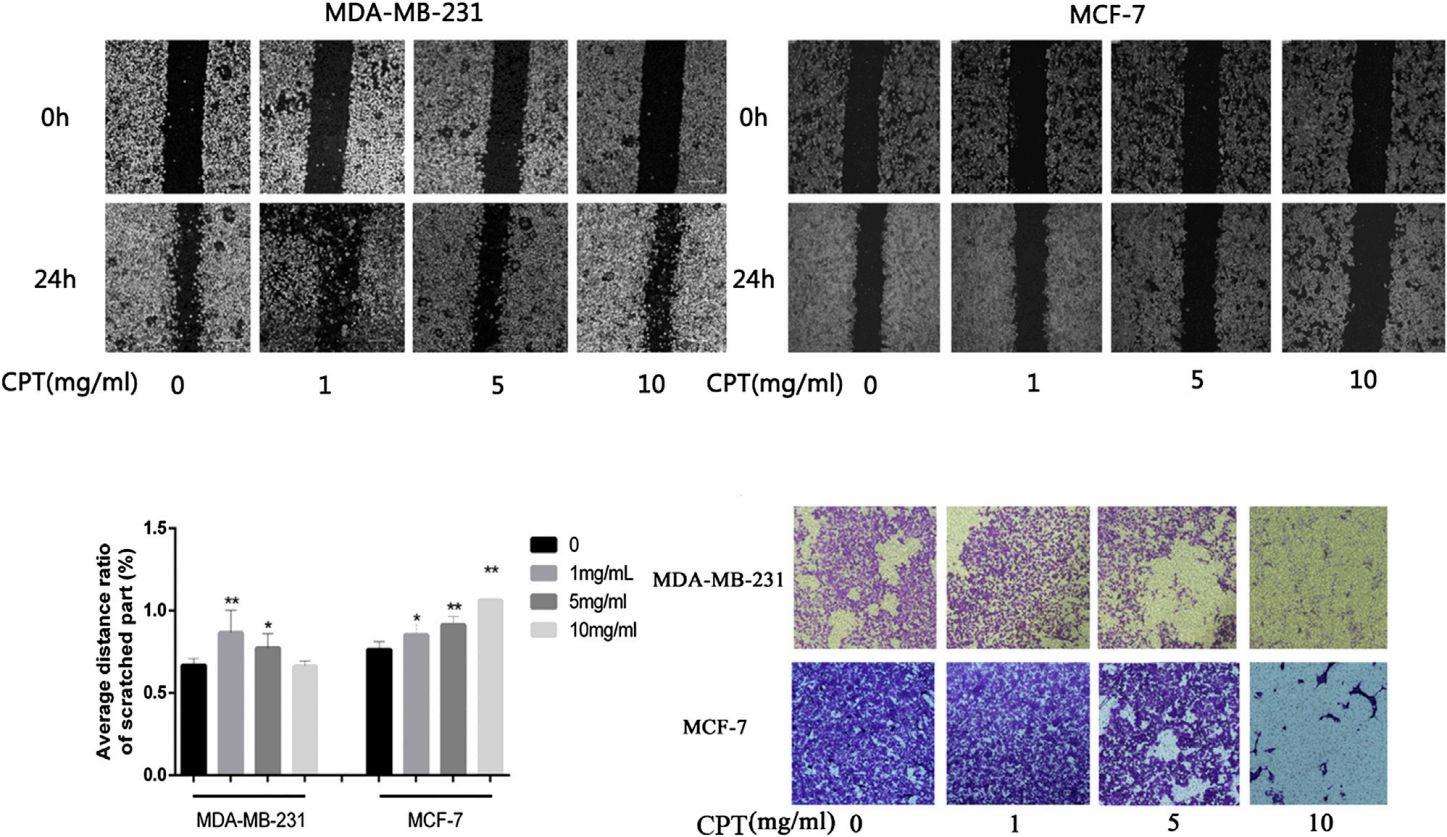
The results of cell migration and transwell invasion experiments. **(A,B)** At the same concentration of CPT, the distance of migration of MCF-7 cells was significantly lesser than that of MDA-MB-231 cells. **(C)** Graph of average distance ratio of scratches. **(D)** as the concentration of CPT was increased (0, 1, 5, 10 mg/ml), its inhibitory effect on the invasive ability of MCF-7 and MDA-MB-231 cells was enhanced. *p < 0.05, **p < 0.01 vs. control (0 mg/ml).

In addition, the results of cell migration and transwell invasion experiments showed that MCF-7 cells were more sensitive to CPT intervention than MDA-MB-231 cells. At the same concentration of CPT, the distance of migration of MCF-7 cells was significantly lesser than that of MDA-MB-231 cells (Figures 4A–C), and this inhibition of migration was concentration-dependent, with increased CPT concentration resulting in stronger the inhibition of migration of the cells. Furthermore, as the concentration of CPT was increased, its inhibitory effect on the invasive ability of MCF-7 and MDA-MB-231 cells was enhanced (Figure 4D).

### Expression of CDK1, ESR1, and CCNA2 in MCF-7 and MDA-MB-231 Cells

**FIGURE 5:**
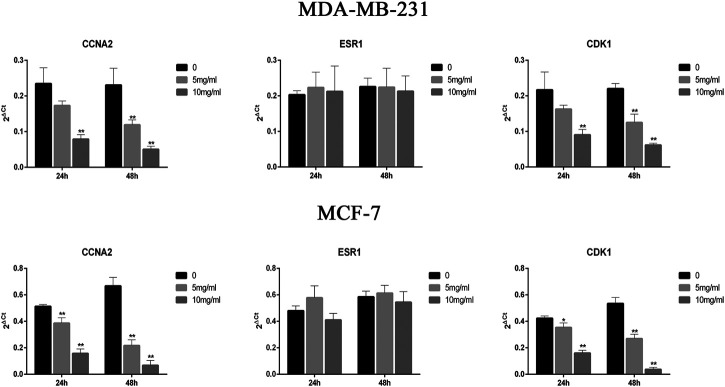
CPT inhibited the expression of *CDK1* and *CCNA2* in MCF-7 cells more significantly then MDA-MB-231. The expression of *ESR1* was not affected by the concentration or duration of CPT treatment in either cell line. MDA-MB-231 **(A)** and MCF-7 **(B)** cell was treated for 24 or 48 h with the indicated concentrations (0, 5, 10 mg/ml) of CPT, levels of *CDK1*, *CCNA2*, and *ESR1* were determined using RT-PCR. *p < 0.05, **p < 0.01 vs. control (0 mg/ml).

To analyze the effect of CPT on the expression of *CDK1*, *ESR1*, and *CCNA2* in MCF-7 and MDA-MB-231 cells, RT-PCR was performed (Figure 5). The results showed that the expression of *CDK1* and *CCNA2* in MCF-7 cells and MDA-MB-231 cells were inhibited at higher concentrations and durations of CPT treatment. Moreover, CPT inhibited the expression of *CDK1* and *CCNA2* in MCF-7 cells more significantly. The expression of *ESR1* was not affected by the concentration or duration of CPT treatment in either cell line.

## Discussion

In BRCA, steroid hormones, mainly estrogens and progestins, are widely recognized as key promoters and/or enhancers (Clarke et al., 1994). About 70% of BRCAs are estrogen receptor positive, and depend on estrogen signaling (Demas et al., 2019). Increased transcription of estrogen receptor target genes promotes cell proliferation and antagonizes apoptosis, contributing to BRCA (Yao et al., 2019). In the past decade, several novel endocrine therapies have been investigated as first-line treatment for hormone receptor-positive BRCA in clinical trials (Clarke et al., 2009). However, resistance to endocrine therapy limits the treatment of many tumors that express estrogen receptor alpha protein (ESR1) (Jones et al., 2019).

Human cells and tissues consist of a complex network of systems with redundant, convergent, and divergent signaling pathways. Malignant and complex systemic diseases generally involve pathological changes in multiple gene products, signaling pathways, and interconnected signaling networks present between cells (Borisy et al., 2003). Network pharmacology provides a novel way to think about drug discovery, which can be used to visualize the potentially complex relationships between multiple components and multiple targets (Tang and Aittokallio, 2014). The use of molecular protein networks provides an opportunity to study potential targets for complex diseases and drug intervention mechanisms (Liu et al., 2019a). The principle of network intervention is especially suitable for the treatment of malignant tumors. The accumulation of cancer genomic data provides the raw material for in-depth research in cancer (Cancer Genome Atlas Network, 2012). In this study, we constructed a multi-layer network by analyzing high-throughput data combined with molecular network visualization methods, including screening differentially expressed disease genes, molecular protein network construction, and identifying drug targets to predict drug targets through a systems biology and network pharmacology approach. Molecular docking techniques are used to predict the affinity between a small drug molecule and a target, directly revealing the interaction between the drug molecule and the target to elucidate their structure-activity relationship (Ferreira et al., 2015). Through a series of screening and validation, we initially identified that CPT may interfere with ER-positive BRCA by regulating *CDK1*, *ESR1*, and *CCNA2*. The discovery of many drugs is usually driven by the search for new molecular targeted therapies. For complex malignant diseases, single-targeted therapies lack the therapeutic flexibility that makes it difficult to correct the disease state (Li et al., 2019). In this study, by combining high-throughput data analysis with network pharmacology techniques, CPT was initially identified as a multi-target drug that could simultaneously stimulate the characteristics of multiple targets in the disease signaling pathway, providing a novel approach for the clinical treatment of BRCA.

Our current experimental data supports the recommendation that ER-positive BRCA cells are sensitive to CPT intervention. As a hormone receptor-positive cell line, MCF-7 has different ER expression than triple-negative MDA-MB-231 (Das et al., 2019). Therefore, we believe that CPT is a potential therapeutic agent for the treatment of ER-positive BRCA. In previous studies of CPT and BRCA, CPT and its synthetic derivatives were used as STAT3 inhibitors to induce tumor cell apoptosis in the BRCA cells MDA-MB-231 (Zhang et al., 2018). In addition, as a multi-target drug, CPT can also signal or induce endoplasmic reticulum stress *via* p38/JNK and Erk1/2 (Xu et al., 2017; Liu et al., 2019b). Therefore, in cell viability and invasion experiments, MDA-MB-231 cells showed sensitivity to CPT intervention, and CPT can be used for ER-negative BRCA, i.e., resistant to drugs such as tamoxifen. In the cell scratch assay, the percentage of wound healing distance between the scratches was significantly lower in MCF-7 cells than in MDA-MB-231 cells after CPT intervention, Combined with the invasion experiment, it was shown that CPT can inhibit the migration of MCF-7, indicating that CPT may be involved in inhibiting the metastasis of ER-positive BRCA, And the latest research found that through different ways to affect and regulate the tumor microenvironment, the natural drug small molecule CPT has an anti-metastatic effect, and the mechanism of action needs further investigation.

ESR1 regulates the expression of estrogen receptors. Estrogen and its receptors are involved in the regulation of gene expression, and affect cell proliferation and differentiation in target tissues (Stein and Yang, 1995). As a key regulator of the cell cycle, *CDK1* promotes G2-M conversion, regulates G1 progression and G1-S conversion by binding to multiple interphase cyclins. CDK1 is an effective therapeutic target for inhibitors in cancer therapy (Wang et al., 2011). *CCNA2*, which controls the G1-S and G2-M transitions in the cell cycle, interacts with *CDK1* and *CDK2* throughout the cell cycle and forms a specific serine/threonine protein kinase holoenzyme complex with them, and activates them (Pagano et al., 1992). In our study, CPT did not significantly inhibit or promote *ESR1*. Previous studies have shown that CPT can bind to *ESR1* and affect the activation of downstream pathways. The downstream pathway of ESR1, such as the PI3K-AKT-mTor signaling pathway, is associated with growth, proliferation, differentiation, and apoptosis of BRCA cells. Activation of PI3K-AKT-mTor signaling pathway can promote BRCA cell proliferation and protein synthesis and is an important target for cancer therapy (Simoncini et al., 2000; Engelman, 2009). CPT inhibits *CDK1* and *CCNA2*, and the inhibitory effect is stronger in the ER-positive MCF-7 cells. Extracellular signal-regulated kinase MAPK3 is involved in a variety of cellular processes such as proliferation, differentiation, regulation of inflammatory responses, cytoskeletal remodeling, cell movement and invasion, through increased production of matrix metalloproteinases (Buonato and Lazzara, 2014). *MAP2K1* phosphorylates and activates *MAPK3*, while *CDK1* is involved in phosphorylation and activation of *MAP2K1*, and *CCNA2* is involved in the activation of *CDK1* (Pagano et al., 1992; Inselman and Handel, 2004; Wortzel et al., 2015). CPT may inhibit the proliferation of BRCA cells by inhibiting the activation and signaling of MAPK signaling pathway by inhibiting *CDK1* and *CCNA2*. Interestingly, the intervention of CPT inhibited *CDK1* and *CCNA2* expression differently in the ER-positive BRCA cells. The precise molecular mechanism of this differential effect needs further studies, and may be related to the ER-positive status of the cells although the distribution of tumor genes in the cell lines is similar. Overall, our data support that CPT is a potential drug for the treatment of ER-positive BRCA.

In this study, through high-throughput data analysis, network dimensionality reduction, molecular docking, and *in vitro* experimental verification models, we initially obtained the molecular intervention mechanism of CPT on ER+ BRCA. This provides effective insights and ideas for the research on the intervention of natural medicine small molecules on complex diseases. However, our research results still need further experimental data and clinical data to support in order to better understand the molecular intervention mechanism of CPT on the proliferation and metastasis of ER+ BRCA.

## Conclusion

In conclusion, our findings support that BRCA is sensitive to CPT, especially ER-positive BRCA. CPT inhibits the proliferation and migration of ER-positive BRCA cells by regulating *CDK1*, *CCNA2*, and *ESR1*. Therefore, CPT can be used as a potential drug for the treatment of ER-positive BRCA, providing new insights for its clinical treatment. More importantly, this study used a combination of high-throughput data analysis and network pharmacology as an effective tool for discovering the molecular mechanisms of a natural product in cancer therapy.

## Data Availability Statement

The datasets presented in this study can be found in online repositories. The names of the repository/repositories and accession number(s) can be found in the article/Supplementary Material.

## Author Contributions

CS, HL, and CG conceived and designed the study; QL, LL, JZ, and JY performed data analysis; CL, CZ, and FF contributed analysis tools; HL and CG wrote the paper.

## Funding

This work is supported by the grants from National Natural Science Foundation of China (81673799, 81973677, 81703915).

## Conflict of Interest

The authors declare that the research was conducted in the absence of any commercial or financial relationships that could be construed as a potential conflict of interest.

## Abbreviations

BP, biological process; BRCA, breast cancer; CC, cell component; CCNA2, cell cycle egg A2; CDK1, cyclin-dependent kinase 1; CPT, cryptotanshinone; DEGs, differentially expressed genes; ER, Estrogen receptor; ESR1, estrogen receptor; GEO, Gene Expression Omnibus; GO, gene ontology; KEGG, Kyoto Gene and Genomic Encyclopedia; MF, molecular functions; RT-PCR, RT-PCR.
